# Socially mediated polyandry: a new benefit of communal nesting in mammals

**DOI:** 10.1093/beheco/aru143

**Published:** 2014-08-28

**Authors:** Yannick Auclair, Barbara König, Anna K. Lindholm

**Affiliations:** Institute of Evolutionary Biology and Environmental Studies, University of Zurich, Winterthurerstrasse 190, CH-8057 Zurich, Switzerland

**Keywords:** cooperation, mammals, maternal care, maternal defense, multiple mating, *Mus musculus domesticus*.

## Abstract

In many species, females have evolved behavioral strategies to reduce the risk of infanticide. For instance, polyandry can create paternity confusion that inhibits males from killing offspring they could have sired. Here, the authors propose that females could socially obtain the same benefits by nesting communally. Singly sired litters could be perceived as a large multiply sired litter once pooled together in a single nest. Long-term data from a wild house mouse population showed that monandrous litters (singly sired) were more common in communal than in solitary nests and 85% of them were raised with litters sired by different males hence becoming effectively polyandrous (multiply sired). These socially polyandrous litters had significantly higher offspring survival than genetically or socially monandrous litters and reached a similar survival to that of multiply sired litters raised in solitary or communal nests. Furthermore, the number of sires within nests significantly improved offspring survival whereas the number of mothers did not. These results suggest that the survival benefits associated with communal nesting are driven by polyandry and not communal defense. This socially mediated polyandry was as efficient as multiple paternity in preventing infanticide, and may also occur in other infanticidal and polytocous species where the caring parent exhibits social behavior.

## Lay Summary

Communal nesting allows social females to avoid infanticide in much the same way as multiple mating: through paternity confusion. Litters sired by a single male can be perceived as a large multiply-sired litter once pooled together in a communal nest. This socially mediated polyandry was as efficient as multiple paternity in preventing infanticide. The number of mothers in communal nests did not explain offspring survival hence rejecting the communal defense hypothesis.

## INTRODUCTION

Infanticide, the act of killing parental care dependent conspecific non-offspring, is reported in a wide range of taxa, and is perpetrated by both males and females (Hausfater and Hrdy 1984; Hoogland 1985). For the perpetrator, infanticide may be accidental (e.g., offspring crushed by fighting adults), provide food resources, reduce resource competition, reduce future competitors of own offspring, prevent adoption or, for males, potentially reduce other males’ reproductive success while increasing and hastening their access to victimized females (Ebensperger 1998). Females typically bear extensive reproductive costs compared to males, and thus are likely to have evolved several counter-strategies to minimize loss of reproductive investment (Agrell et al. 1998).

Females can reduce infanticide by their mating behavior through polyandry, by mating with multiple partners during a single reproductive event (Hrdy 1979). Polyandry can provide females with access to resources and indirect (genetic) fitness benefits (Hosken and Stockley 2003; Parker and Birkhead 2013), but may also reduce the vulnerability of offspring to infanticidal males. Polyandrous females give multiple males a perceived reproductive contribution, which creates paternity confusion that inhibits those males from killing what could be their own offspring (van Schaik et al. 2000; Wolff and MacDonald 2004). In the bank vole *Myodes glareolus*, a species in which females do not gain resources from males and in which infanticide is a heritable behavioral strategy (Mappes 2012), recent experiments showed that recruitment was improved when females mated multiply with all resident males compared to multiple matings that included only 1 resident male (Klemme and Ylönen 2010).

An alternative route for females to reduce infanticide is to prevent conspecifics that represent a threat from accessing their offspring through increased nest defense (Agrell et al. 1998). However, survival and social constraints (e.g., searching for food, territory defense) can limit the time that females can spend on nest defense. Consequently, females might engage in a cooperative strategy and nest communally, allowing offspring defense duties to be shared and potentially reducing the time the young are left alone. Cooperative interactions of that kind do not require kinship between the partners to evolve, although it may favor more stable relationships, as they are mutually beneficial (Clutton-Brock 2002; Bshary and Bergmüller 2008). For instance, in lions *Panthera leo* unrelated females form coalitions that are more successful in protecting offspring than a single female alone (Packer and Pusey 1983). Similarly, an increased nest defense has been suggested to explain why communally nesting female house mice *Mus musculus domesticus* benefit from higher rates of offspring survival compared to solitarily nesting females who raise their litters alone (Manning et al. 1995). Convincing evidence for cooperative female defense of communal nests, however, is still missing in this species.

In the present study, we propose a novel hypothesis to explain why offspring survival is higher in communal nests. Communal nesting allows females to pool their litters in a nest with those of females that mated with different males. Singly sired litters could thus be perceived as a large multiply sired litter once together in a communal nest, and infanticide could thereby be reduced for all the litters in the nest. We therefore tested the hypothesis that communal nesting allows females to socially obtain the offspring survival benefits associated with paternity confusion. To that end, we used long-term data from a free-roaming population of wild house mice *Mus musculus domesticus*.

The house mouse is a highly territorial species where reproductive competition is pronounced (Oakeshott 1974; König and Lindholm 2012) and favors infanticide (vom Saal et al. 1995). Females are known to raise their litters solitarily or communally by grouping their pups in a single nest (Weidt et al. 2014) in which they share maternal care (König 1997). Polyandry is a common reproductive strategy within this species that results in multiply sired litters (Dean et al. 2006; Firman and Simmons 2008; Manser et al. 2011) but its influence on infanticide is currently unknown. We measured polyandry through multiple paternity in solitarily reared litters or in the pooled litters from communal nests, and communal nesting through multiple maternity of pooled littermates. Then, we tested whether polyandry and communal nesting improved offspring survival. We accounted for population density as a predictor of the intensity of the intrasexual reproductive competition, a factor that can favor infanticide (Ebensperger 1998; Mappes 2012).

## METHODS

### Study population and reproductive activity

Data were collected from a wild house mouse population open to dispersal but closed to predators established in a 70 m^2^ former agricultural building outside of Zurich, Switzerland (König and Lindholm 2012). Mice are subject to predation from cats, foxes, and birds of prey when they leave the building. Food, a 50/50 mixture of oats and hamster food (Landi AG, Switzerland), and water were provided *ad libitum* to avoid enhancing infanticide (Mappes 2012). This setup represents a natural habitat for mice, a species which is commensal with humans and establishes populations where there is easily accessible food (Berry 1970; Cucchi et al. 2002; Pocock et al. 2004). Adult population density was estimated every 7 weeks by capturing the entire population. Matings cannot be controlled in this population as it would require removing individuals from their territories to laboratory conditions. Females and males were therefore free to choose their mating partner(s) and females could choose to nest solitarily or communally. Reproduction occurred in 40 nest boxes in which we systematically searched for new litters approximately every 10 days from January 2007 to December 2009. All litters were documented, and pup age was estimated based on morphological development. Skin pigmentation, development of the ears, growth of the fur, teeth eruption, and eye development give reliable cues about the age of the pups (±1 day, day of birth was considered as day 1) (König and Lindholm 2012). This study includes litters found in the first 3 days of life. Animal use and experimental design were approved by the Veterinary Office Zürich, Switzerland (Kantonales Veterinäramt Zürich, no. 215/2006).

### Pup survival

We searched intensively for every documented litter when the pups were expected to be 13-days old and used survival to 13 days as a proxy for survival until the onset of weaning. Although weaning starts at 17 days (König and Markl 1987), 13-days old is the closest age to weaning where we can handle litters without disturbance as pups open their eyes and become mobile on the 14th day (König and Lindholm 2012). Pup survival was defined by the difference in litter size between the first (age 1–3 days) and second census (age 13 days). Pups that were missing at the second census were considered deceased. Previous studies have reported that pups killed by infanticide typically present bites on their head, neck, or stomach or miss their body parts (Huck et al. 1982; Labov et al. 1985; Manning et al. 1995). Among the 254 pup corpses that were not desiccated when we found them over the course of this study, 77.1% showed at least one such type of injury. There was no sign of injury on only 1.2% of these corpses. Information was missing for the remaining 21.7%. The 3 most common injuries observed in our population were the absence of a body part (38.6%), the presence of bites or open wounds (31.5%), and a hole in the skull (23.2%). Infanticide is so common in this population that it can sometimes be observed directly.

### Genetic analyses: mother identity and number of sires

We extracted DNA from tissue samples taken from ears of pups found on the 13th day of age and from all adults as well as from pup corpses following the procedure detailed in Auclair et al (2014). A parentage analysis of these samples using 25 microsatellite loci (Auclair et al. 2014) provided the identity of the mother as well as the number of sires within litters to a 95% level of confidence using Cervus 3.0 (Kalinowski et al. 2007). Litters were categorized as genetically monandrous when they were sired by a single male and genetically polyandrous when they were sired by more than 1 male. Both paternity and maternity were successfully assigned to 146 litters produced by 106 females. An additional 143 litters disappeared entirely and were not accounted for in these analyses as no genetic material was available. There was no significant difference in the proportion of these that were solitary (*N* = 72) versus communal (*N* = 71) (χ^2^ = 0.01, df = 1, *P* = 0.933).

### Communal versus solitary nests

Communal nests were defined as those containing litters from more than 1 mother, which was visually obvious only when pups in the nest differed in age. Therefore, we confirmed maternity using results of the genetic analyses which allowed us to identify 56 solitary litters and 90 communal litters.

### Genetically versus socially polyandrous litters

The full parentage assignment of each litter provided the cumulated number of different sires within solitary and communal nests. Genetically monandrous litters raised in communal nests were categorized as genetically monandrous but socially polyandrous whenever they were associated with a litter sired by at least 1 different male.

### Statistical analyses

All statistical tests were performed using R 3.0.2 (R Development Core Team 2013). Univariate and multivariate statistical analyses were used to examine pup survival. In the univariate analysis, pup survival was first categorized with respect to communal nesting and polyandry and then tested with independent chi-square tests (Table 1). In the multivariate analysis, pup survival was set as the response variable in a generalized linear mixed model fitted with a binomial error distribution and corrected for over-dispersion. Mother identity was included as a random factor to control for non-independence of repeated measures from the same individuals. The fixed effects structure included communal nesting (measured as the number of different mothers within the nest), polyandry (measured as the number of different sires within the nest), pup age (at first census), population density, the 2 interactions involving communal nesting with polyandry and population density, and the interaction between polyandry and population density. The significance of the fixed terms was given by Wald’s tests. Following the recommendations of Nakagawa and Schielzeth (2013), we provide a full summary statistic of this model (Table 2).

**Table 1:** Summary table of the independent chi-square tests used to compare offspring survival with respect to communal nesting and polyandry

Comparison	χ^2^	*P*
Solitary genetically monandrous vs. Solitary genetically polyandrous	4.20	0.040
Solitary genetically monandrous vs. Communal genetically monandrous	0.86	0.354
Solitary genetically monandrous vs. Communal genetically polyandrous	9.70	0.002
Solitary genetically monandrous vs. Communal genetically monandrous but socially polyandrous	10.43	0.001
Solitary genetically polyandrous vs. Communal genetically monandrous	8.74	0.003
Solitary genetically polyandrous vs. Communal genetically polyandrous	1.17	0.278
Solitary genetically polyandrous vs. Communal genetically monandrous but socially polyandrous	1.45	0.229
Communal genetically monandrous vs. Communal genetically polyandrous	16.02	<0.001
Communal genetically monandrous vs. Communal genetically monandrous but socially polyandrous	16.95	<0.001
Communal genetically polyandrous vs. Communal genetically monandrous but socially polyandrous	0.01	0.905

**Table 2:** Full statistics of the mixed effect modeling of pup survival

	Null model	Full model	Wald *Z*	*P*
Fixed effects	*b* [95% CI]	*b* [95% CI]		
Intercept	1.36 [0.91, 1.81]	−2.27 [−5.95, 1.41]	−1.21	0.228
Communal nesting (# mothers/nest)	—	0.42 [−1.95, 2.79]	0.34	0.730
Polyandry (# sires/nest)	—	2.64 [0.48, 4.80]	2.40	0.016
Pup age	—	0.46 [−0.04, 0.96]	1.80	0.071
Population density	—	−0.01 [−0.05, 0.03]	−0.48	0.630
Communal nesting (# mothers/nest): Polyandry (# sires/nest)	—	−0.36 [−0.62, −0.10]	−2.71	0.007
Communal nesting (# mothers/nest): Population density	—	0.01 [−0.01, 0.03]	0.59	0.555
Polyandry (# sires/nest): Population density	—	−0.01 [−0.03, 0.01]	−1.08	0.278
Random effects	VC	VC		
Mother identity	1.530	1.468		
Observations (correction for over-dispersion)	2.830	2.219		
Residuals	—	—		
Fixed effects	—	1.685		
*R* ^2^_GLMM(m)_	—	19.45%		
*R* ^2^_GLMM(c)_	—	62.02%		

The intercept of the full model represents a litter reared by 1 mother, sired by 1 male, found when 1-day old, at a population density of 44 adults.

—, not applicable/available; CI, confidence interval; GLMM, generalized linear mixed model; *R* ^2^_GLMM(c)_, conditional *R* ^2^ for GLMM (i.e., variance explained by fixed and random factors); *R* ^2^_GLMM(m)_, marginal *R* ^2^ for GLMM (i.e., variance explained by fixed factors); VC, variance components.

## RESULTS

### Polyandry and communal nesting

Although litters from communal nests were smaller than litters from solitary nests (communal litters: 3.31±0.23 pups [mean ± SE] − range 1–8, solitary litters: 5.32±0.27 pups − range 1–12; Student *t-*test: *t*_144_ = −5.64, *P* < 0.001), they were sired by a similar number of males (communal litters: 1.37±0.06 sires − range 1–4, solitary litters: 1.43±0.08 sires − range 1–3; Wilcoxon test: *W* = 2360.5, *P* = 0.439).

**Figure 1:**
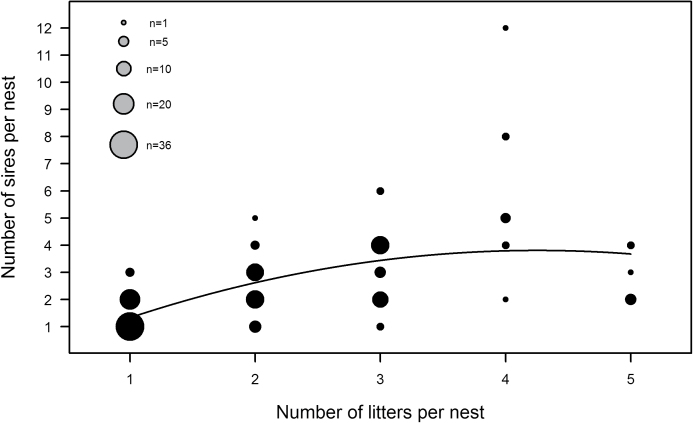
Positive correlation between the number of litters per nest and the number of different sires per nest. Figure shows observed data and regression line (*R* ^2^ = 0.36, *P* < 0.001).

The number of different sires within a nest showed a curvilinear increase with the number of litters pooled together (*R* ^2^ = 0.36; *F*_2,143_ = 41.31, *P* < 0.001; Figure 1). Communal nests, which consisted of 2.85±0.10 litters on average, summed more than twice as many sires as solitary nests (communal nests: 3.08±0.18 sires − range 1–12, solitary nests: 1.43±0.08 sires − range 1–3; Wilcoxon test: *W* = 4293, *P* < 0.001).

**Figure 2:**
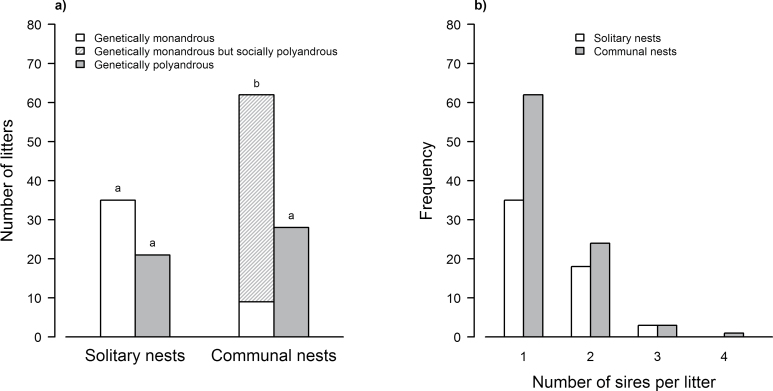
Distributions of (a) the litters according to communal nesting and polyandry (indices above columns (a, b) refer to significant differences between groups of data), and (b) the number of sires per litter within solitary and communal nests.

Genetically polyandrous litters were as common in solitary nests as in communal nests (χ^2^ = 1.00, df = 1, *P* = 0.317; Figure 2a,b), whereas genetically monandrous litters were more often observed in communal nests than in solitary nests (χ^2^ = 7.51, df = 1, *P* = 0.006; Figure 2a,b). There was no significant difference between the number of genetically monandrous and genetically polyandrous litters raised in solitary nests (χ^2^ = 3.50, df = 1, *P* = 0.061; Figure 2a).

### Univariate analysis of pup survival

Pup survival was significantly higher in communally raised litters than in solitarily raised litters (communal litters: 79.71±4.23% [mean ± SE_p_ (binomial standard error)], solitary litters: 56.71±6.62%; χ^2^ = 3.88, df = 1, *P* = 0.049). Similarly to communal nesting, genetic polyandry (multiple paternity within litters) also significantly improved pup survival with polyandrous litters having a greater pup survival than monandrous litters (polyandrous litters: 81.39±2.68%, monandrous litters: 46.54±5.75%; χ^2^ = 9.49, df = 1, *P* = 0.002).

**Figure 3:**
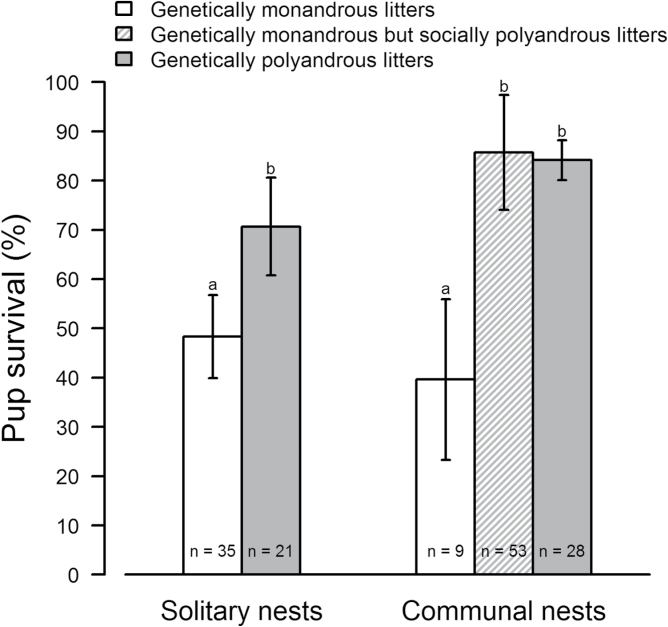
Influence of polyandry and communal nesting on pup survival (observed data ± SE_p_). Indices above columns (a, b) refer to significant differences between groups of data.

Genetically polyandrous litters raised in solitary nests had a similar pup survival to those raised in communal nests (Table 1; Figure 3). The same was observed between genetically monandrous litters raised in solitary nests or in communal nests. Genetically polyandrous litters, however, showed a higher pup survival than genetically monandrous litters, both within solitary nests and within communal nests. Socially polyandrous litters raised in communal nests showed a greater pup survival than that of genetically monandrous litters raised in communal nests as well as in solitary nests, and similar to that of genetically polyandrous litters regardless of whether they were raised in communal or in solitary nests.

### Multivariate analysis of pup survival

**Figure 4:**
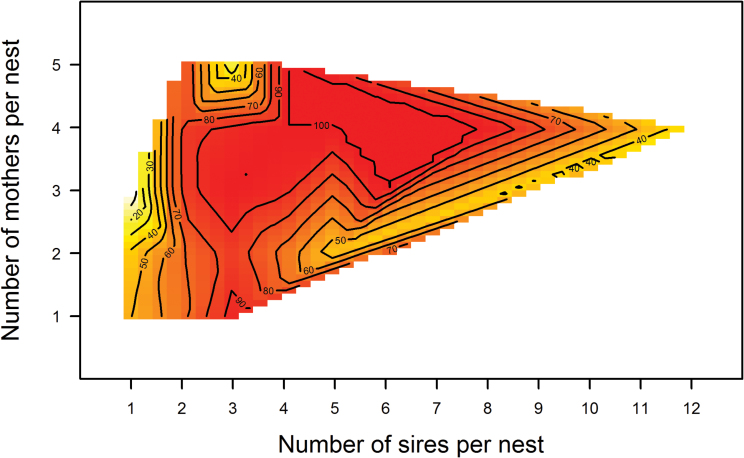
3D representation of the interaction between the number of sires and the number of mothers within a nest on pup survival (isoclines represent pup survival in %; color increases gradually from yellow to red with increasing pup survival).

There was a significant interaction between the number of sires and the number of mothers (Table 2; Figure 4). A greater number of sires within a nest significantly improved pup survival whereas the number of mothers alone had no significant effect (Table 2).

Although litters from communal nests were found when they were older than litters from solitary nests (communal litters: 2.30±0.09 days [mean ± SE], solitary litters: 1.91±0.11 days; *t*_144_ = 2.65, *P* = 0.009), the age at which the pups were found had no significant effect on pup survival (Table 2). Population density and its 2 interactions with polyandry and the number of mothers per nest had no significant effect on pup survival (Table 2).

## DISCUSSION

Communal nesting and multiple paternity of litters both improved offspring survival in wild house mice. However, we showed that nests containing offspring sired by multiple males survived better than nests containing offspring sired by a single male, both in solitary and communal nests. Mating with multiple males allows polyandrous females to confuse the paternity of their litters hence preventing males from committing infanticide as they could kill their own offspring (Perrigo et al. 1990; van Schaik et al. 2000; Wolff and MacDonald 2004; Klemme and Ylönen 2010). We therefore suggest that paternity confusion is the mechanism responsible for the higher offspring survival associated with communal nesting. If males are unable to recognize their young ones, but refrain from attacking offspring in a nest attended by a female with which they previously mated, then nests in which there are more potential fathers are better protected. A female that has not mated polyandrously would benefit from socially mediated polyandry, by associating her offspring with offspring of other females that mated with different males to herself, in a communal nest.

Our hypothesis would be falsified if: 1) offspring mortality does not reflect infanticide, 2) males could discriminate their offspring from others within a communal nest, 3) polyandry does not result in multiple paternity, 4) females within a communal nest had the same mating partners, and 5) communal nesting is not the result of an active choice. Each of these points, however, can be addressed.

1) As the population studied here was not exposed to nest predation, postnatal offspring mortality could only be explained by postpartum cannibalism by the mother, abandonment, disease, or infanticide. Both postpartum cannibalism and abandonment are expected to occur very soon after birth so that mothers avoid wasting energy in costly maternal care such as lactation (Hammond and Diamond 1992). Because we first found litters when they were on average 2-days old, postpartum cannibalism and abandonment are not very likely to explain offspring mortality in our population. Although restricted food availability has been shown to promote cannibalism of older pups by lactating females (König 1989b), this should not occur in our population as food was provided *ad libitum*. Moreover, nearly all pups we found looked healthy (were of normal appearance and did not show deformities). Infanticide, on the other hand, is very common in rodents and other taxa (Hrdy 1979). For instance, a field study showed that 51% of the litters suffered infanticide in black-tailed prairie dogs *Cynomys ludovicianus* (Hoogland 1985). Since most of the dead pup corpses we collected displayed injuries typical of infanticide and since infanticide was also occasionally directly observed, we think that most of the offspring mortality observed in our population resulted from infanticide. Previous studies suggested that the presence of conspecifics is a better predictor of juvenile mortality than the presence of predators which is considered as insignificant (Berry 1970; Berry and Bronson 1992; Brown 1953; Southwick 1955). Consequently, the substantial effect that predation could have on offspring survival is not very likely to mask the benefits of polyandry.
2) There is little evidence that males can recognize their own offspring in altricial birds and mammals (Blaustein et al. 1987; Kempenaers and Sheldon 1996; Mateo 2003). In the house mouse, the most convincing evidence of male kin recognition showed that males use prior matings with a female, more particularly those that included ejaculation, to assess their affiliation with her offspring (Huck et al. 1982; McCarthy and vom Saal 1986; Perrigo et al. 1989, 1990). Female house mice also have very limited, if any, ability to recognize their own offspring (König 1989a, 1989b; Hager and Johnstone 2005).
3) The category of “singly sired” litters will include females that mated with only 1 male as well as those that mated multiply, especially when litter sizes are small (Neff et al. 2002). The number of different sires within litters is likely to underestimate the actual frequency of multiple mating as there is a high postcopulatory competitive skew between the males in house mice (Levine 1967; Dean et al. 2006). In our study, we assume that females with multiply sired litters had more mating partners than those with singly sired litters.
4) We found that only 10% of the litters found in communal nests were pooled with litters sired by the same male. Thus, nearly all communal nests could benefit from paternity confusion.
5) Communal nests in the study population are the result of female choice. Females choose among available nesting partners (Weidt et al. 2014). Experimental evidence shows that when females that prefer each other initiate a communal nest, reproductive success is higher than in nests of females that had no preference for each other (Weidt et al. 2008). Here, we found a higher proportion of females whose litters were singly sired in communal nests compared to solitary nests, raising the possibility that these females may actively try to socially acquire the benefits of polyandry.

Taken together, the evidence is convincing that our new hypothesis can be applied to communal nesting in wild female house mice. Firstly, we demonstrated that females benefit from polyandry. This may explain why multiple paternity was similar among females raising their offspring in solitary or communal nests. Moreover, the prevalence of multiple paternity renders the widespread assumption of a polygynous mating system in house mice questionable (Latham and Mason 2004). Secondly, polyandry can explain the offspring survival advantage associated with communal nesting. Communal nesting can benefit monandrous females by reducing the risk from male infanticide through socially mediated polyandry when litters are pooled with others sired by different males. Furthermore, females who have the option to communally nest could potentially avoid costs of polyandry (Clutton-Brock and Parker 1995; Stockley 1998, Parker and Birkhead 2013) and mate with their preferred male.

It has been proposed that more females sharing a communal nest could correlate with an increased nest defense (see Manning et al. 1995). For this hypothesis to hold true, all litters from communal nests should show a better offspring survival than those from solitary nests. Our data, however, do not support this hypothesis as illustrated in Figures 3 and 4. Furthermore, we found a significant interaction between the number of females and the number of sires within a nest, indicating that the offspring survival benefits of polyandry are modified by the number of females at the nest, pointing to a complex role of social structure in offspring survival. Previous studies have demonstrated the limited influence of maternal aggression in preventing infanticide perpetrated by male house mice, hence suggesting that paternity confusion may be more efficient against male infanticide (Palanza et al. 1995). As an alternative to an increasing number of females who could defend a nest, offspring survival may be improved through a better coordination between these females. Relatedness does not only help stabilizing the relationship between cooperative partners, but it can also improve the outcome of their interaction (Holmes and Sherman 1982). A recent study, however, showed that relatedness between communally nesting females had no effect on patterns of nest attendance or the time their litters are left alone (Auclair et al. 2014).

Previous hypotheses for communal nesting include the nonadaptive hypothesis of misdirected maternal care as a by-product of social living (Pusey and Packer 1994; Hayes 2000; but see Weidt et al. 2014), and the hypotheses that communal nesting is adaptive via offspring thermoregulation benefits (Hayes and Solomon 2006), improved offspring milk intake and growth rate (Sayler and Salmon 1969; Mennella et al. 1990; Heiderstadt and Blizard 2011), better offspring immunity (Roulin and Heeb 1999; Boulinier and Staszewski 2008), and higher female reproductive success (König 1994). We provide here a new adaptive hypothesis for the evolution of communal nesting where females who produced litters sired by a single male can improve the survival of their offspring by pooling their litters with others sired by different males, which we call socially mediated polyandry. This socially mediated polyandry was as efficient as genetic polyandry in improving offspring survival. Our new hypothesis may not only apply to the other species known for providing communal care to their offspring which represents 15% of all mammalian species in more than 7 orders (Eisenberg 1981). Any polytocous vertebrate producing litters of more than 1 offspring, in which infanticide is a behavioral strategy, and where the caring parent exhibits social behaviors could also be a good candidate.

## FUNDING

This work was supported by the University of Zurich, the Swiss National Science Foundation (31003A-120444), and the Stiftung für Wissenschaftliche Forschung an der Universität Zürich (F-74310-07-01).
